# The Class III Peroxidase gene *TaPRX-2A* controls grain number per spike in common wheat (*Triticum aestivum* L.)

**DOI:** 10.3389/fpls.2024.1501029

**Published:** 2025-02-06

**Authors:** Dongtian Zang, Wenjia You, Yangyang Wu, Pengyue Wang, Zhiyu Wang, Qingyun Yang, Shatong Chi, Peisen Su

**Affiliations:** ^1^ College of Agriculture and Biology, Liaocheng University, Liaocheng, China; ^2^ Institute of Huanghe Studies, Liaocheng University, Liaocheng, China

**Keywords:** wheat, peroxidases (PRXs), TaPRX-2A, grain number per spike (GNS), co-expression analysis

## Abstract

Some peroxidases (PRXs) are involved in abiotic stress response. However, to the best of our knowledge, the effects of PRXs on agronomic traits including grain number per spike (GNS), spikelet number per spike (SNS) and spike length (SL) are also largely unknown. In our study, we cloned a wheat PRX gene *TaPRX*-2A and identified its function in controlling GNS by generating transgenic overexpression lines. The results showed that *TaPRX-2A* overexpression displayed lower GNS and shorter SL, compared with the wild-type plants. RNA-seq analysis indicated alterations in various pathways including flavonoid biosynthesis, lignin biosynthesis, phytohormone signaling, as well as sucrose and starch biosynthesis. Co-expression analysis showed that transcription factors, such as bHLH, WRKY, and bZIP may be involved in the regulation of various genes associated with these pathways. Our findings provide insights into the mechanisms by which PRXs regulate agronomic traits, illustrating potential applicability in crop improvement programs.

## Introduction

Common wheat (*Triticum aestivum* L.) is a major cereal crop worldwide, providing ~20% of dietary calories to humans and constitutes 30% of global grain production (Shiferaw et al., 2013; Chand, 2009). Yield in wheat is a complex quantitative trait determined by three components: kernel weight, spike number per unit area, and grain number per spike (GNS) (Ma et al., 2018). Spike length (SL) is another crucial component primarily affecting GNS (Wu et al., 2014). Up to now, numerous genes, such as *TaSPL17*, *TaAIRP2-1B*, *TaSus1*, *TaWRKY37-A1*, *TaMYC2-A1*, *TaMYB30-A1* have been identified that regulate GNS and SL in wheat (Liu et al., 2023; Wu et al., 2017; Zhang et al., 2023; Lin et al., 2024)

PRXs are antioxidant enzymes that catalyze the oxidation of many substrates and are widespread in several organisms, such as plants, animals, and microbes. PRXs are structurally categorized into nonhaem and haem (animal and nonanimal) (Welinder, 1992; Taurog, 1999). The PRX superfamily is functionally divided into three classes: I (e.g., APXs), II (lignin PRXs), and III (secretory PRXs) (Bhatt and Tripathi, 2011). Several studies have reported the class III families in plants to be multigenic. For example, 374, 159 and 169 class III PRXs exist in *Triticum aestivum*, *T*. *urartu* and *Aegilops tauschii*, respectively (Yan et al., 2019). Additionally, 119 members in maize, 75 class in *Arabidopsis thaliana*, 151 in *Brachypodium distachyon* and 155 in rice have been identified (Fawal et al., 2013).

In recent years, numerous class III PRXs have been functionally annotated, with many of them being involved in tolerance to various adverse stresses, especially abiotic stresses (drought and salt), and pathogen defense (Cosio and Dunand, 2009; Su et al., 2020, Su et al., 2023). For example, the overexpression of *OsPrx30* enhanced the susceptibility of rice to bacterial blight by reducing H_2_O_2_ contents (Liu et al., 2021). In *Arabidopsis thaliana*, *AtPrx64* overexpression improved the tolerance to aluminum-induced stress by suppressing ROS accumulation and enhancing lignin content (Wu et al., 2017). In previous study, we found that *TaPRX-2A* overexpression improves drought and salt tolerance in transgenic lines by activating ABA pathway and enhancing oxidative stress tolerance, such as higher antioxidant activities of peroxidase (POD), superoxide dismutase (SOD) and catalase (CAT) enzymes, and reduction of reactive oxygen species (ROS) accumulation, and lower levels of MDA content (Su et al., 2020, Su et al., 2023). Meanwhile, class III PRXs are involved in developmental processes. For example, *AtPrx71* is involved in cell growth and inhibition of cell expansion through H_2_O_2_ accumulation (Raggi et al., 2015). At low temperatures, the apoplastic class III peroxidases *PRX62* and *PRX69* promote root hair growth in *Arabidopsis thaliana* (Pacheco et al., 2022). However, it remains to be elucidated whether PRXs are involved in regulating spike agronomic traits in wheat.

Common wheat is a major cereal crop worldwide. Its yield is determined by KNS, which is associated with SL. In this study, we functionally characterized a class III PRX gene *TaPRX-2A* which regulates SL. *TaPRX-2A* overexpression lines displayed lower GNS and shorter SL, which occurred through activating flavonoids, sucrose and starch biosynthesis as well as phytohormone (ABA, IAA and JA) pathways. Our findings revealing the function of *TaPRX-2A* in regulating GNS can provide researchers with new insights into the class III PRXs-related molecular mechanisms underlying GNS formation in wheat.

## Materials and methods

### Plant materials and growing conditions

The common wheat (*T. aestivum* cultivars “KN199”) and *TaPRX-2A* overexpression transgenic lines were used in this study and obtained from Shandong Agricultural University. The *TaPRX-2A* overexpression transgenic lines and WT “KN199” were as grown in the field at Liaocheng, Shandong, China in 2022 and 2023. Each transgenic line and WT plants were planted in a ten-row plot. Each row was 2.0 m in length with 20 plants, and the row spacing was 10 cm. The spikes from main shoots of 5 individual plants were randomly selected in each transgenic line and WT plants to measure spike length (SL), grain number per spike (GNS), plant height (PH), and thousand kernel

weight (TKW).

### Isolation and cloning of *TaPRX-2A* and transgenic plant generation

*TaPRX-2A* was cloned from the wheat cultivar ‘Sumai 3’. The leaves of seedlings were harvested and ground into a powder in liquid nitrogen. For total RNA extraction, the TRIzol reagent (TransGen, Beijing) was used. It was reverse-transcribed into first-strand cDNA by HiScript II Q RT SuperMix (Vazyme, Nanjing). The full-length cDNA sequence of *TaPRX-2A* was obtained from Ensembl plants (http://plants.ensembl.org/index.html ; ID: TraesCS2A02G573900). We designed the gene-specific primers based on the *TaPRX-2A* sequence using Primer 5. Subsequently, *TaPRX-2A* was cloned into the vector PC414C and then ligated into the OE vector PC186 (pUbi::GWOE::Nos). The resulting vector was used to transform plants of the wheat cultivar ‘KN199’ using particle gun-mediated gene transformation (Yao et al., 2006).

### Transcriptional profiling

The spikes of *TaPRX-2A* overexpression transgenic lines and WT plants at booting stage were harvested and immediately placed in liquid nitrogen for transcriptome analysis. The total RNA were extracted with RNAprep Pure Plant Kit (TIANGEN), then were sent to Metware Corporation (Wuhan) for transcriptional profiling. The transcriptome analysis was performed by Illumina HiSeq™ 2000 instrument. We removed the adapter sequences and low sequencing quality reads using Fastp (Chen et al., 2018). The paired-end reads from transcriptome sequencing data were mapped onto reference genome of wheat (http://plants.ensembl.org/Triticum_aestivum/Info/Index) by HISAT2 (Kim et al., 2015), then were assembled into transcripts using StringTie (Pertea et al., 2015). We normalized the expression data for each gene by the featureCounts (Liao et al., 2014). Then, we analyzed differentially expressed genes (DEGs) using the DEGseq R package with the default parameters (false discovery rate (FDR) < 0.05, |log_2_(fold change)|>= 1). Gene Ontology (GO) enrichment and Kyoto Encyclopedia of Genes and Genomes (KEGG) enrichment analysis of DEGs were performed by online website (https://www.genome.jp/kegg/). We performed the analysis of GO terms and KEGG pathways with the default parameters (Q-values <0.05).

### Gene co-expression networks

To better elucidate functional relationships of DEGs in flavonoid biosynthesis, lignin biosynthesis, phytohormone signaling, sucrose and starch biosynthesis, we constructed protein interaction network of the Inter- and intra-pathways and the visualization of gene co-expression networks were performed through software Cytoscape (Shannon et al., 2003).

### Co-expression pattern cluster and transcription factor binding site analysis

To explore the binding sites of TFs to the promoters of the genes involved in flavonoid biosynthesis, lignin biosynthesis, phytohormone signaling as well as sucrose and starch biosynthesis, 2000 bp sequences upstream of the initiation codon were retrieved. The binding sites of the TFs were analyzed using the TBtools software (Chen et al., 2020). The co-expression pattern cluster of the TFs and these genes were analyzed utilizing R (base package). We grouped the DEGs with the same expression trends into a data set by analyzing the variations in the patterns of mRNA expression abundance among the *TaPRX-2A* overexpression lines and WT plants. The data set was then drawn.

### Expression pattern analysis

The leaves of “Sumai 3” were harvested from seedling and ground into powder in liquid nitrogen for RNA extraction. We used the TRIzol reagent (TransGen, Beijing) to extract the total RNA. The total RNA was reversed into First strand cDNA by HiScript II Q RT SuperMix (Vazyme, Nanjing), which was used as the template for expression analysis of these genes in different pathways. The expression levels of the genes were performed by quantitative real time PCR using the Roche LightCycler ^®^480 system (Roche, Germany). The wheat *18SrRNA* gene was utilized as internal controls for normalization. The quantifications of these genes were calculated using the 2^^(−ΔΔCT)^ method. Each experiment collected at least three independent biological replicates. All the qRT–PCR primers used in this study are listed in **Supplementary Table S4**.

## Results

### Overexpression of *TaPRX-2A* exhibits negative effects on GNS

**Figure 1:**
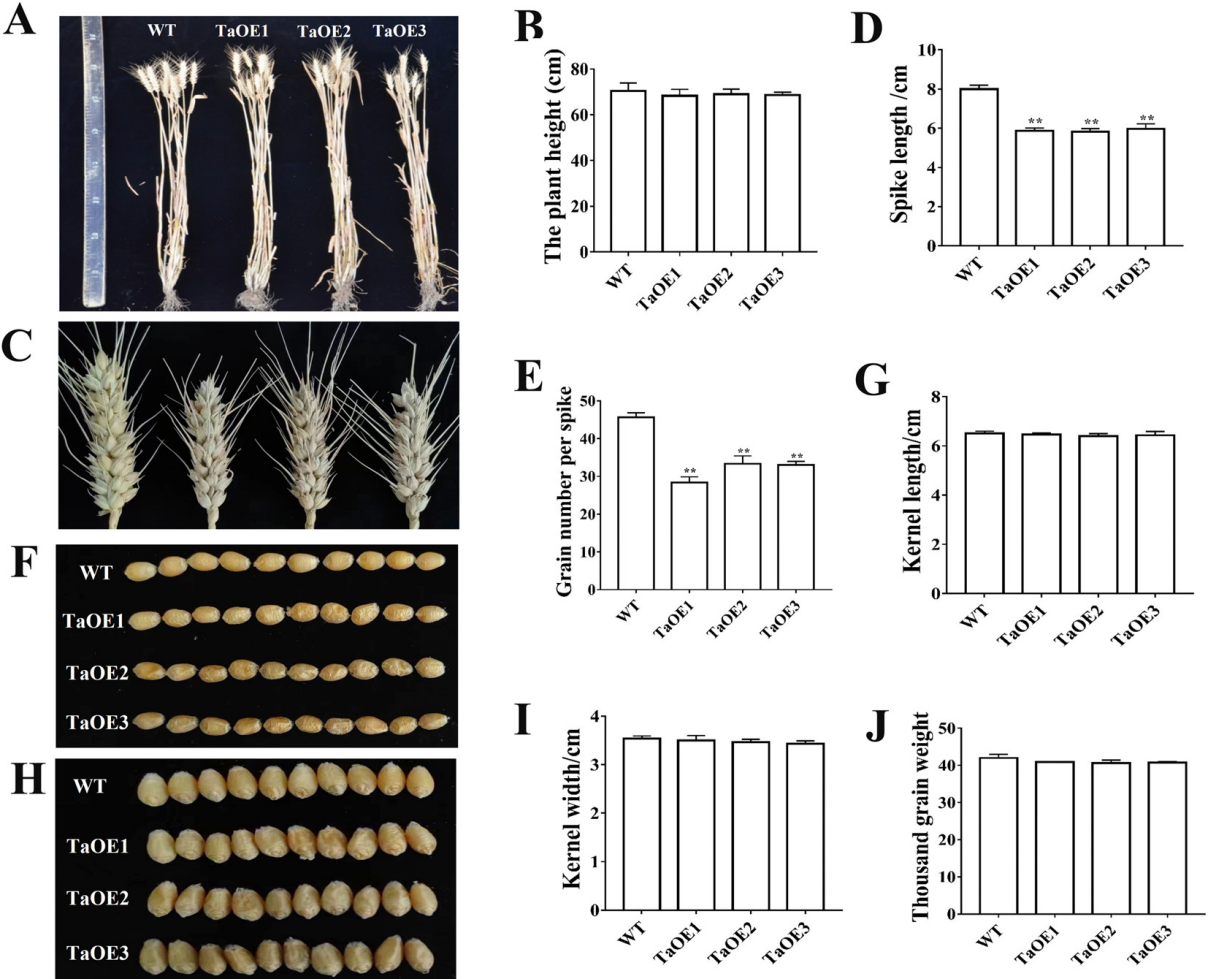
Phenotypic characterization of spike traits in *TaPRX-2A* overexpression transgenic lines and WT plants. **(A)** Representative images of plant height between *TaPRX-2A* overexpression transgenic lines and WT plants. **(B)** Plant height. **(C)** Representative images of spikes between *TaPRX-2A* overexpression transgenic lines and WT plants. **(D)** Spike length (SL). **(E)** Grain number per spike (GNS). **(F-I)** Representative images showing length and width of kernels from *TaPRX-2A* overexpression transgenic lines and WT plants. **(J)** Thousand grain weight. Each experiment was conducted in three replicates and values are means ± SD (n = 6). ** indicates a significant difference at P < 0.01.

In a previous study, we analyzed the expression levels of *TaPRX-2A*, *TaPRX-2B* and *TaPRX-2C* based on transcripts per kilobase million (TPM) values collected from the WheatOmics site (Seifert et al., 2016; Li et al., 2018). *TaPRX-2A* was highly expressed in the double ridge stage (KNIV) and microspore embryogenesis S3 (**Supplementary Figure S1**). To understand the association between the alterations in *TaPRX-2A* expression and spike development, we generated *TaPRX-2A-*overexpression lines in the common wheat ‘KN199’ background and successfully genotyped three independent homozygous lines. The *TaPRX-2A-*overexpression lines and WT plants were planted in a greenhouse and their agronomic traits were observed. Plant height did not differ significantly between the transgenic lines and WT plants (**Figure 1A, B**). The spike morphology differed in SL and GNS between the two. Compared with WT (8 cm), the SL (5.89 cm) was substantially shortened in the transgenic lines (**Figure 1C, D**) and the GNS (31.25) decreased markedly (45.6) (**Figure 1E**). These agronomic traits, including kernel length, kernel width, and thousand-grain weight did not vary conspicuously between the transgenic lines and the WT (**Figures 1F–J**). These results confirmed the function of *TaPRX-2A* in regulating GNS.

### *TaPRX-2A* overexpression alters gene expression profiles in wheat

**Figure 2:**
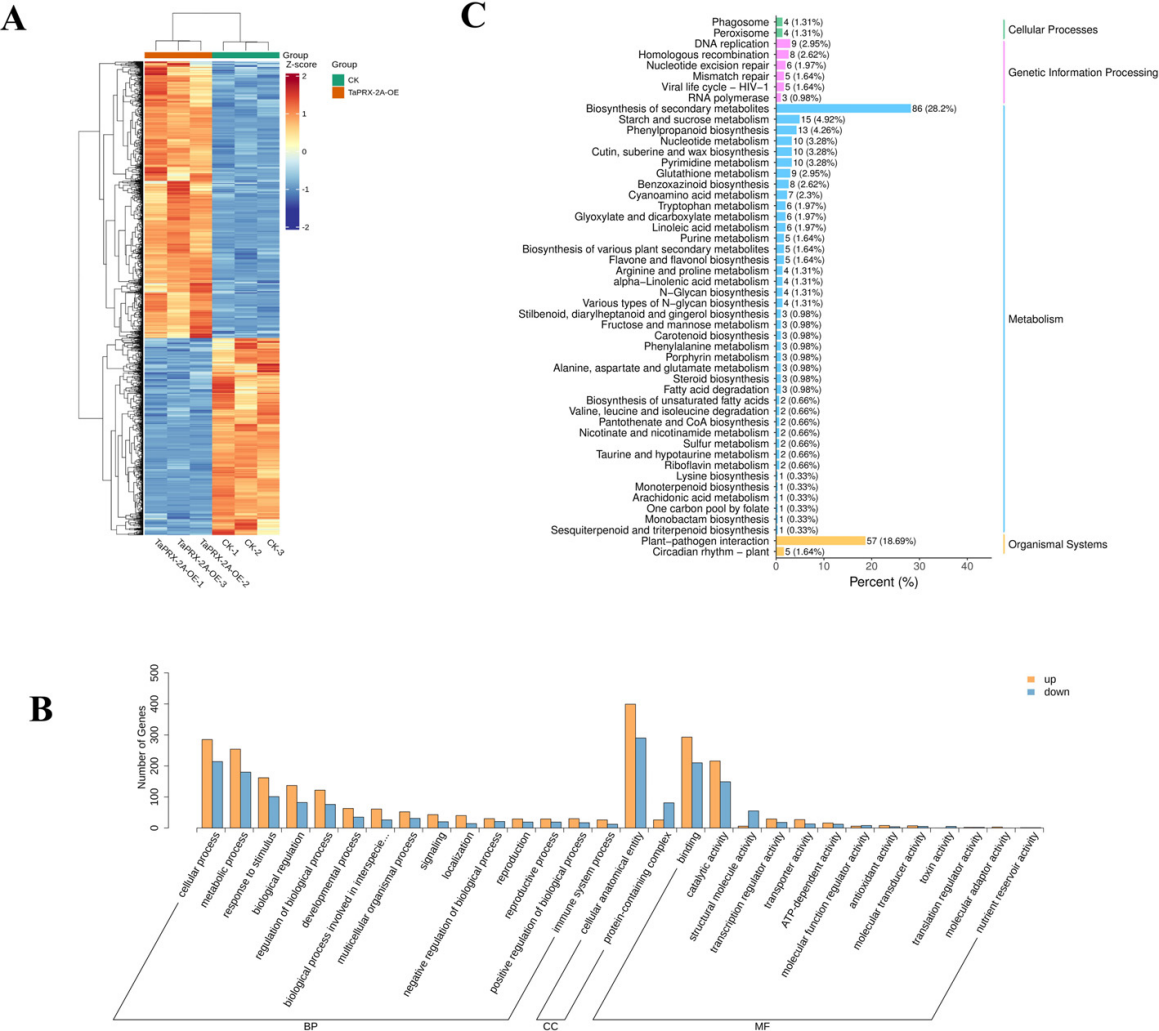
Transcriptome analysis in *TaPRX-2A* overexpression transgenic lines and WT plants. **(A)** The heatmap of the DEGs between *TaPRX-2A* overexpression transgenic lines and WT plants. The heatmap was created based on FPKM expression values. **(B)** GO classification of DEGs between *TaPRX-2A* overexpression transgenic lines and WT plants. **(C)** KEGG pathway enrichment between *TaPRX-2A* overexpression transgenic lines and WT plants. Each sample was conducted in three replicates and named “CK-1”, “CK-2”, and “CK-3”.

To further elucidate the regulatory mechanisms by which *TaPRX-2A* affects GNS in wheat, the transcriptomes of *TaPRX-2A* overexpression lines and WT plants were compared. We found 1317 DEGs between the two, including 548 down and 769 upregulated ones (**Figure 2A**, **Supplementary Figure S2**, **Supplementary Table S1**). In addition, GO classification and KEGG analyses revealed that biological processes (biological regulation, response to stimulus, developmental processes, growth and reproductive processes), molecular functions (catalytic, antioxidant, binding, and structural molecule activities) and cell components (protein-containing complex and cellular anatomical entity) were highly enriched terms (**Figure 2B**, **Supplementary Table S2**). KEGG enrichment analyses indicated the DEGs to be mainly enriched in hormone signal transduction, starch and sucrose metabolism, phenylpropanoid biosynthesis, and MAPK signaling pathway (**Figure 2C**, **Supplementary Table S2**).

### Enriched DEGs in TaPRX-2A overexpression lines in flavonoid and lignin biosynthesis pathways

**Figure 3:**
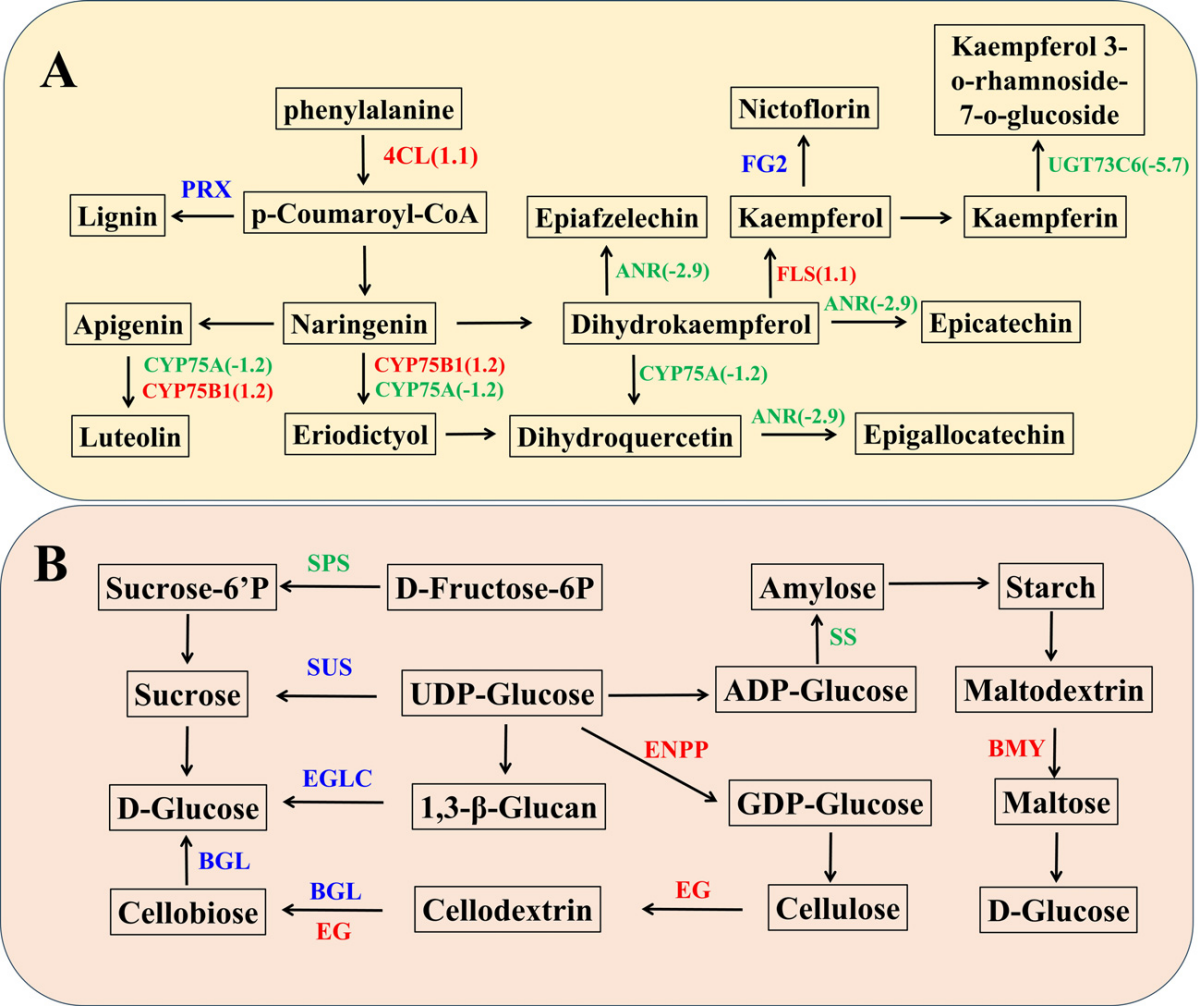
*TaPRX-2A* overexpression alters flavonoid, sucrose and starch biosynthesis pathway. **(A)** The network was constructed based on known genes expression levels involved in flavonoid pathway. **(B)** The network was constructed based on known genes expression levels involved in sucrose and starch biosynthesis pathway. The blue color represents gene families encoding the same group of proteins with similar functions, where individual genes within these families show either up-regulation or down-regulation, while red represented upregulated and green represented downregulated in *TaPRX-2A* overexpression transgenic lines compared to WT plants, respectively. *4CL*, 4-coumarate–CoA ligase; *FLS*, flavonol synthase; *CYP75B1*, flavonoid 3’-monooxygenase; *ANR*, anthocyanidin reductase; *UGT73C6*, flavonol-3-O-L-rhamnoside-7-O-glucosyltransferase; *CYP75B1*, flavonoid 3’-monooxygenase; *ENPP*, ectonucleotide pyrophosphatase; *BMY*, beta-amylase; *EG*, endoglucanase; *SPS*, sucrose-phosphate synthase; *SS*, starch synthase; *BGL*, beta-glucosidase; *EGLC*, glucan endo-1,3-beta-D-glucosidase.

We generated a network illustrating the relationships between *TaPRX-2A*, flavonoid and lignin biosynthesis to further study how *TaPRX-2A* affects GNS. The expression levels of 19 genes associated with the flavonoid and lignin biosynthesis pathway were altered, among which 4-coumarate-CoA ligase (*4CL*), flavonol synthase (*FLS*), and flavonoid 3’-monooxygenase (*CYP75B1*) were upregulated compared to the WT plants, while anthocyanidin reductase (*ANR*), flavonoid 3’,5’-hydroxylase (*CYP75A*), and flavonol-3-O-L-rhamnoside-7-O-glucosyltransferase (*UGT73C6*) were downregulated (**Figure 3A**, **Supplementary Table S3**). In addition, the expression profiles of PRX (TraesCS6D02G054600(1.79326532777333), TraesCS2A02G573900(3.1764958969293), TraesCS6A02G047400(1.22826501558943), TraesCS2B02G613900(1.08348514467672), TraesCS6D02G127100(1.10257940548889), TraesCS3B02G209700(1.63944763210169), TraesCS6D02G108400(1.11318941408978), TraesCS5A02G323200(-1.05843591801709), TraesCS5D02G330300(-1.27076575042592), TraesCS7A02G452900(-1.44407938464799), TraesCS3B02G578000(-1.19525771883893)) and flavonol-3-O-glucoside (*FG2*) (TraesCS4A02G385500(1.71126108560809), TraesCS5A02G325200(-1.24485547304725)) were differed. Some genes are upregulated in the overexpression lines, including TraesCS6D02G054600, TraesCS2A02G573900, TraesCS6A02G047400, TraesCS2B02G613900, TraesCS6D02G127100, TraesCS3B02G209700, TraesCS6D02G108400, TraesCS4A02G385500, while others are downregulated, such as TraesCS5A02G323200, TraesCS5D02G330300, TraesCS7A02G452900, TraesCS3B02G578000, TraesCS5A02G325200 (**Supplementary Table S3**). In addition, the expression levels of the flavonoid and lignin biosynthesis pathways genes were measured, which supported the transcriptome data set (**Supplementary Figure S3**). Detailed information is displayed in **Supplementary Table S3**.

### Enriched DEGs in TaPRX-2A overexpression lines in sucrose and starch biosynthesis pathways

*TaPRX-2A* overexpression modulated the sucrose and starch biosynthesis pathway. The transcriptomic data revealed significant differences in the expression profiles of many sucrose and starch biosynthesis-related genes. Among them, ectonucleotide pyrophosphatase (*ENPP*), beta-amylase (*BMY*) and endoglucanase (*EG*) were upregulated, while sucrose-phosphate synthase (*SPS*) and starch synthase (*SS*) were downregulated compared to the WT plants (**Figure 3B**, **Supplementary Table S3**). In addition, the expression profiles of sucrose synthase (*SUS*) (TraesCS4B02G167500(1.27482309742693), TraesCS4D02G169800(-6.32054333672797)), β-glucosidase (*BGL*) (TraesCSU02G036600(1.05689194722632), TraesCS4D02G038500(9.09766571668372), TraesCS2B02G550300(-1.17328950677694), TraesCS2B02G401500(-1.47248308622032)) and glucan endo-1,3-β-D-glucosidase (*EGLC*) (TraesCS6D02G099100(1.1146060158768), TraesCS4D02G191900(-1.14398141299911)) were varied. Some genes are upregulated in the overexpression lines, including TraesCS4B02G167500, TraesCSU02G036600, TraesCS4D02G038500, TraesCS6D02G099100, while others are downregulated, such as TraesCS4D02G169800, TraesCS2B02G550300, TraesCS2B02G401500, TraesCS4D02G191900 (**Supplementary Table S3**). In addition, the expression levels of these sucrose and starch biosynthesis pathway-associated genes were ascertained, which supported the transcriptome data set. Detailed information is displayed in **Supplementary Table S3**.

### Enriched DEGs in TaPRX-2A overexpression lines in hormone-related pathways

**Figure 4:**
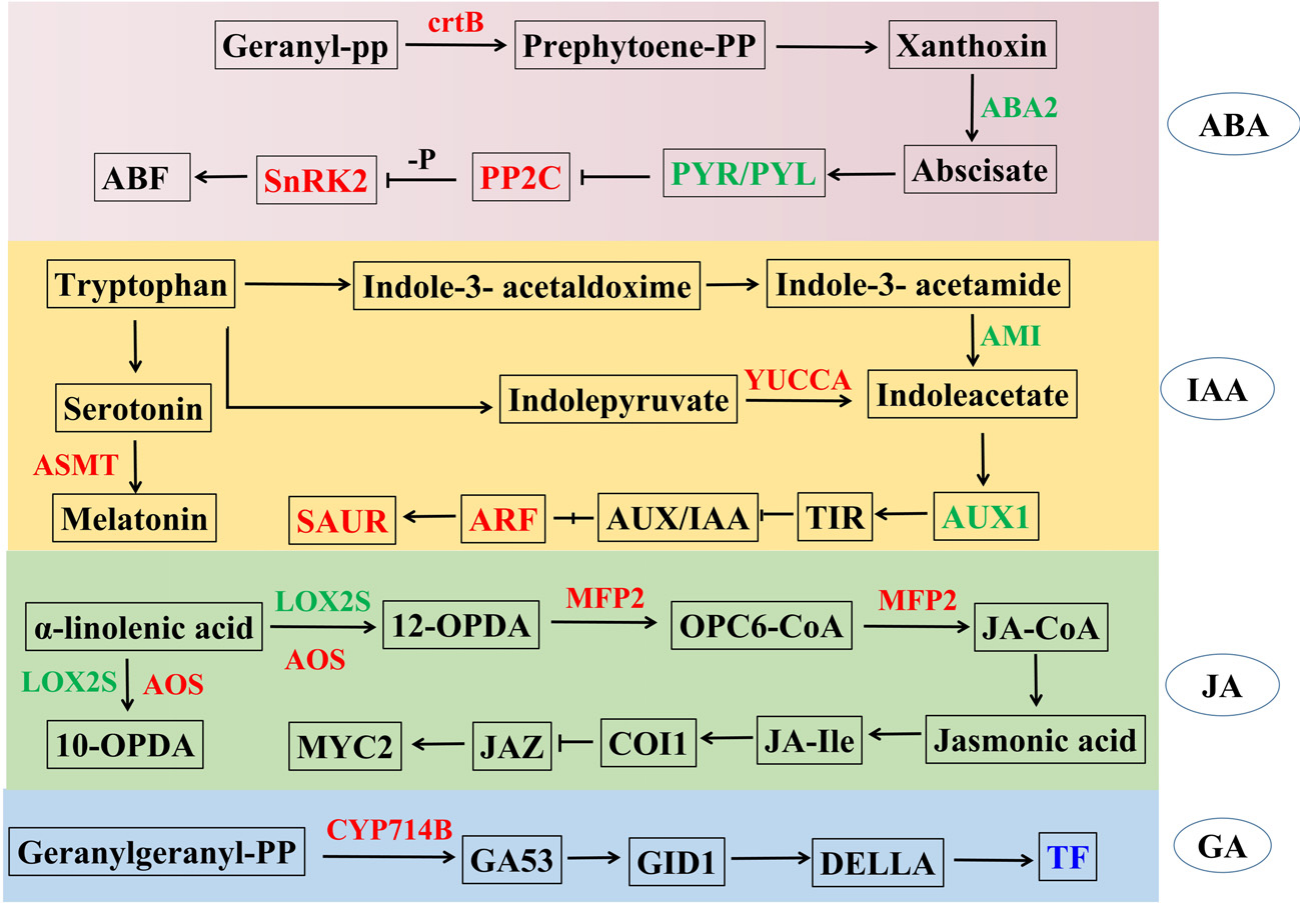
*TaPRX-2A* overexpression alters plant hormone pathways including ABA, IAA, JA and GA. The network was constructed based on known genes expression levels involved in plant hormone pathways. The blue color represents gene families encoding the same group of proteins with similar functions, where individual genes within these families show either up-regulation or down-regulation, while red represented upregulated and green represented downregulated in *TaPRX-2A* overexpression transgenic lines compared to WT plants, respectively. *ctrB*, 15-cis-phytoene synthase; *ABA2*, xanthoxin dehydrogenase; *PP2C*, protein phosphatase 2C; *PYL*, abscisic acid receptor PYR/PYL family; *SnRK2*, serine/threonine-protein kinase SRK2; *YUCCA*, indole-3-pyruvate monooxygenase; *ASMT*, acetylserotonin O-methyltransferase, *AUX1*, auxin influx carrier; *ARF*, auxin response factor; *SAUR*, SAUR family protein; *LOX*, lipoxygenase; *AOS*, hydroperoxide dehydratase; *MFP2*, enoyl-CoA hydratase/3-hydroxy acyl-CoA dehydrogenase; *IF*, phytochrome-interacting factor; *CYP714B*, gibberellin 13-oxidase.

Our transcriptome data indicated that *TaPRX-2A* overexpression also modulated hormone pathways, including ABA, IAA, JA and GA. Therefore, we generated a network to explore the relationships between *TaPRX-2A* overexpression and hormone-associated pathways. The results showed that several genes in the plant hormone pathways were enriched: six ABA-related genes were identified, among which 15-cis-phytoene synthase (*ctrB*), protein phosphatase 2C (*PP2C*) and serine/threonine-protein kinase SRK2 (*SnRK2*) were upregulated in the transgenic lines, while abscisic acid receptor PYR/PYL family (*PYR/PYL*) and xanthoxin dehydrogenase (*ABA2*) were downregulated compared to the WT plants. Further, ten auxin-related genes were found, among which indole-3-pyruvate monooxygenase (*YUCCA*), acetylserotonin O-methyltransferase (*ASMT*), auxin response factor (*ARF*) and SAUR family protein (*SAUR*) were upregulated, while amidase (*AMI*) and auxin-responsive protein (AUX/IAA) were downregulated in the transgenic lines compared to the WT plants. In addition, five JA-related genes lipoxygenase (*LOX*), hydroperoxide dehydratase (*AOS*), *MFP2* and *MYC2*, as well as five GA-related genes (*CYP714B* and TF) were altered upon *TaPRX-2A* overexpression. The detailed information is displayed in **Figure 4** and **Supplementary Table S3**.

To further elucidate the relevant functional relationships, the gene–gene relationships focusing on the flavonoid and lignin biosynthesis, sucrose and starch biosynthesis as well as hormone-related pathways upon *TaPRX-2A* overexpression were analyzed employing STRING (http://string-db.org) (**Supplementary Figure S4**). These genes encompassed *4CL*, CAT, *ABA2*, *MFP2*, *BGL*, *PP2C*, *EG*, *ANR*, *PP2C*, *PYR/PYL*, *AOS*, *LOX*, *GST*, *CYP75A*, *CYP75B1*, *SUS*, *SPS*, *SS* and *ctrB*. They were involved in the same biosynthesis pathway and interacted with each other. For example, *CYP75A* (TraesCS4A02G446400) with *CYP75B1* (TraesCS6D02G015200); *BGL* (TraesCS4D02G038500, TraesCS2B02G550300 and TraesCS2B02G401500) with *EG* (TraesCS6A02G093200 and TraesCS6D02G112100); and *AOS* (TraesCS6D02G172200) with *LOX* (TraesCS5D02G013400) and TraesCS5B02G006500). In addition, the crosstalk between different pathways was also elucidated, among which *MFP2* (TraesCS6D02G116200) in the JA pathway interacted with *ABA2* (TraesCS2B02G143900 and TraesCS2D02G124800) in the ABA pathway, *CAT* (TraesCS6D02G048300) in the antioxidant pathway and *4CL* (TraesCS6D02G141700) in the flavonoid pathway. *ANR* (TraesCS2D02G483100) in the flavonoid pathway interplayed with *ctrB* (TraesCS5D02G365100) in the ABA pathway. The protein–protein interactive relationships are detailed in **Supplementary Figure S4**.

### DEGs related to flavonoid, phytohormon, sucrose and starch biosynthesis pathways are more abundant in *TaPRX-2A* overexpression lines

**Figure 5:**
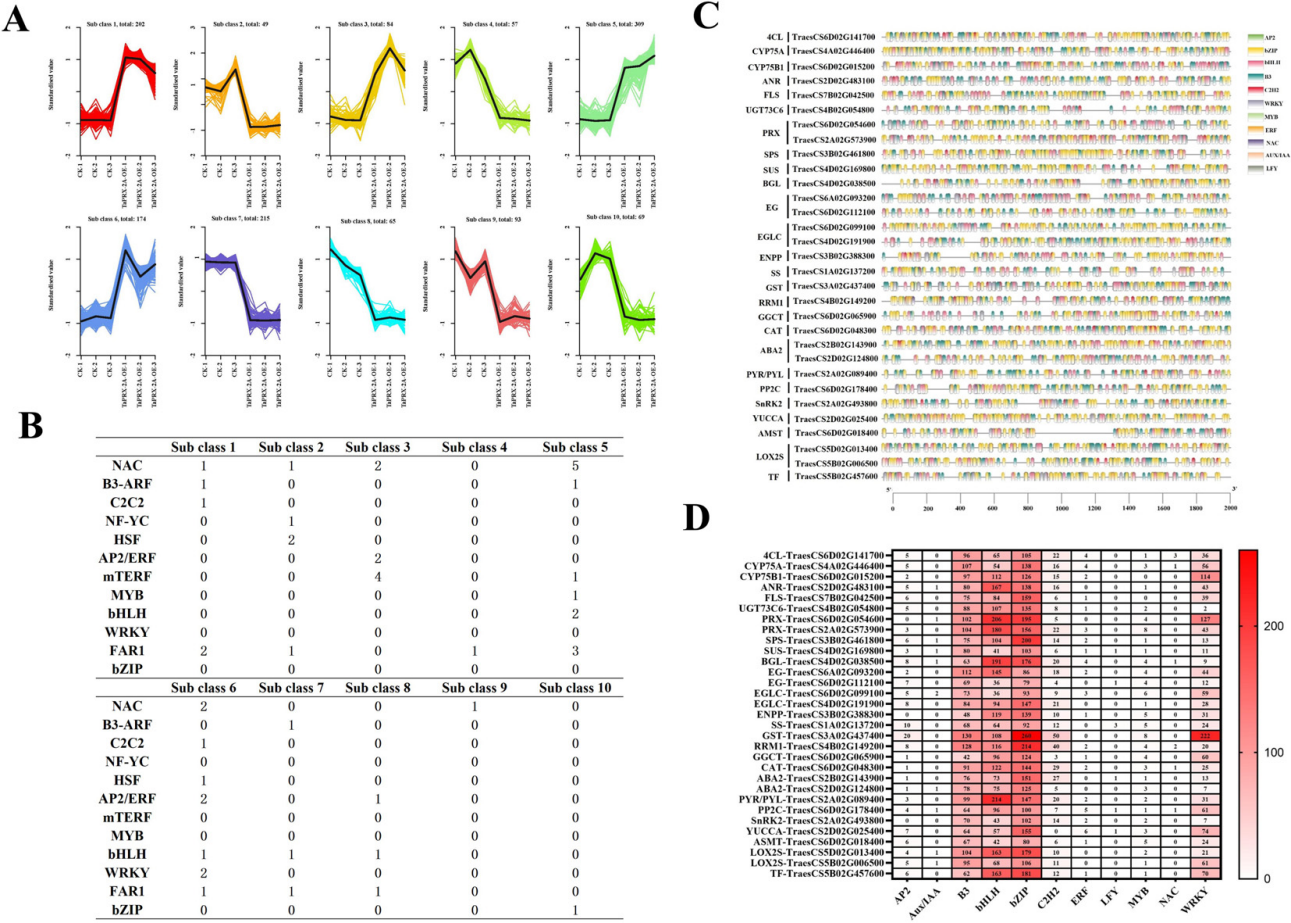
**(A)** The cluster analysis of co-expression patterns of all DEGs. **(B)** Quantity statistics of the transcription factors in different subclasses. **(C)** Promoter binding site analysis of these genes in flavonoid biosynthesis, lignin biosynthesis, phytohormone, sucrose and starch biosynthesis pathways. **(D)** The cis-elements distribution of genes in flavonoid biosynthesis, lignin biosynthesis, phytohormone, sucrose and starch biosynthesis pathways. The upstream (2000 bp DNA sequence) of these genes in different pathways was used to analyze IF binding site and were represented with different colors.

To further elucidate the regulatory relationships between the different pathways, we performed a co-expression cluster analysis of the genes associated with them and all differentially expressed TFs. The Fragments Per Kilobase of exon model per Million mapped fragments (FPKM) values of all DEGs were standardized using R language functions (scale ()), and then cluster analysis was carried out. We divided all DEGs into ten clusters based on different expression profile (**Figure 5A**). Further analysis of the various types of TFs in the different clusters showed that NAC, WRKY, bHLH, FAR1, AP2/ERF and MYB were more abundant in *TaPRX-2A* overexpression lines (**Figure 5B**). The promoter binding sites (sequences 2000 bp upstream of the start site) of these genes from various pathways were analyzed. Compared to the promoters of *4CL*, *CYP75A*, *CYP75B1*, *ANR*, *UGT73C6*, *FLS*, *PRX*, *SPS*, *SUS*, *BGL*, *EG*, *EGLC*, *ENPP*, *SS*, *GST*, *RRM1*, *GGCT*, *CAT*, *ABA2*, *PP2C*, *PYR/PYL*, *SnRK2*, *YUCCA*, *AMST*, *LOX*, and *TF*, more binding sites were identified for *B3*, *bHLH*, *WRKY*, *C2H2*, and *bZIP* (**Figure 5C, D**). *TaPRX-2A* overexpression enhanced the expression of these genes belonging to different pathways by regulating the expression of TFs, such as *B3*, *bHLH*, *WRKY*, and *bZIP*.

## Discussion

Common wheat (*Triticum aestivum* L.) is a major cereal crop worldwide. Its yield is determined by three components: kernel weight, spike number per unit area, and GNS (Ma et al., 2018). SL is a crucial component of yield and primarily affects KNS (Wu et al., 2014). So far, a few wheat SL and GNS-related genes, such as *TaSPL17*, *TaAIRP2-1B*, *TaSus1*, *TaWRKY37-A1*, *TaMYC2-A1* and *TaMYB30-A1* have been identified (Liu et al., 2023; Wu et al., 2017; Zhang et al., 2023; Lin et al., 2024). Here, we functionally characterized the role of the class III PRX gene, *TaPRX-2A* in controlling wheat GNS by activating various metabolic pathways.

GNS is a critical agronomic trait that affects wheat yield strongly. Several studies have reported the control of GNS by various genes. For example, triple mutants of the wheat sucrose synthase gene *TaSus1* displayed lower than the wild-type plants (Shen et al., 2024). The overexpression of *TaSPL17* enhanced GNS and SL as indicated by a high−resolution genotype–phenotype map in wheat (Liu et al., 2023). A mutation in the TF bZIPC and upon interaction with FT2 reduced the SNS in tetraploid wheat (Glenn et al., 2023). *TaMYC2-A1* and *TaMYB30-A1* are involved in wheat spike development as evidenced by integrated multiomics, transcriptional networks, GWAS, and genetic analyses (Lin et al., 2024). However, research on the function of the wheat *PRX* in the context of GNS and SL has been limited. This study validated the effects of *TaPRX-2A* by the phenotypic analysis of newly generated overexpression transgenic lines. The results indicated that *TaPRX-2A* overexpression displayed lower GNS and shorter SL. Further study will be explored the effect of *TaPRX-2A* mutation on GNS and provide materials for crop gene pyramiding molecular breeding.

Extensive research has reported the functional role of starch and sucrose biosynthesis pathways as GNS determinants. For example, the wheat sucrose synthase gene *TaSus1* controlled grain number per spike by influencing the fructose contents. The result showed that expression levels of *TaSus-A1* was higher than *TaSus-B1* or *TaSus-D1* during early carpel development and two single-nucleotide polymorphisms in *TaSus-A1* contributed differently to GNS (Shen et al., 2024). Additionally, suppressed ABA signal transduction reduced grain number by promoting sucrose use in wheat under drought conditions (Zhang et al., 2020). The MATE transporter GFD1 in rice controls the grain-filling duration, grain size and number by interacting with two sugar transporters, OsSWEET4 and OsSUT2 (Sun et al., 2023). These studies suggest that starch and sucrose biosynthesis is involved in wheat spike development. They are consistent with the present findings, wherein *TaPRX-2A* overexpression activated the expression levels of genes including *SPS*, *SUS*, *EGLC*, *ENPP*, *EG*, *SS* and *BGL* involved in the starch and sucrose biosynthesis pathway. Overall, its potential application for wheat improvement to explore the contribution of *TaPRX-2A*, *TaPRX-2B*, and *TaPRX-2D*, interaction between PRXs and starch and sucrose biosynthesis-related genes, and examine different haplotypes of *TaPRX-2A*.

Some study showed that PRX enzymes regulate the polymerization of lignin monomers (Liu et al., 2018). From example, the previous study showed that ZmWRKY86 regulated the peroxidase gene *ZmPRX1* to control drought tolerance by promoting root development and lignification in maize (Zhai et al., 2024). Overexpression of swpa4 peroxidase increases the lignin content in sweet potato (Kim et al., 2008). Consistent with these studies, we also found that *TaPRX-2A* overexpression alters the lignin biosynthesis and expression levels of some WRKYs. In addition, our previous research found that *TaPRX-2A* overexpression reduced ROS levels by enhancing oxidative stress tolerance, such as superoxide dismutase (SOD), peroxidase (POD) and catalase (CAT) enzymes (Su et al., 2020, Su et al., 2023). In *Arabidopsis*, the class III peroxidases PRX62 and PRX69 promote root hair growth by modulating ROS-homeostasis at low temperature (Pacheco et al., 2022). In our study, *TaPRX-2A* overexpression alters the expression levels of antioxidant-related genes, such as CAT、GST、RRM1. Taken together, we predicted that *TaPRX-2A* may control grain number per spike by WRKYs regulation and controlling ROS levels.

Some previous studies have shown that *FT*s affected SNS. For example, *FT2* overexpression reduced spikelet number in *Brachypodium distachyon* and barley, and its loss-of-function mutations increased SNS (Shaw et al., 2019; Glenn et al., 2022). Consistent with these studies, we identified that *TaPRX-2A* overexpression altered the expression levels of *FT*. These results can be explained by *TaPRX-2A* effectively influencing GNS. In addition, several pieces of evidence indicate the crosstalk between starch and sucrose biosynthesis, flavonoids and hormone-related pathways. For example, the CIRCADIAN CLOCK ASSOCIATED1 (*OsCCA1*) mediated panicle development and tiller growth through strigolactone signaling and sugar sensing (Wang et al., 2020). ABA signal transduction regulates grain number by promoting sucrose catabolism (Zhang et al., 2020). ABA is involved in tuning stem elongation and ear development by collaborating with lignin and flavonoid biosynthesis in maize (*Zea mays* L.) (Gao et al., 2023). Our previous studies found that *TaPRX-2A* conferred drought tolerance in wheat via the ABA pathway. This study also identified that *TaPRX-2A* controlled GNS via the ABA pathway based on transcriptomics data. Thus, our evidence combined with those from previous studies suggests that *TaPRX-2A* regulated drought tolerance and developmental balance by activating the ABA signaling pathway. Further study is warranted to explore the networks of growth regulation and stress response-related crosstalk between *TaPRX-2A* and ABA and elucidate critical links, providing a high potential for application in wheat improvement.

## Conclusion

In this study, we characterized the function of the class III peroxidase gene *TaPRX-2A* in controlling grain number per spike in wheat. The results demonstrated that *TaPRX-2A* overexpression significantly decreased grain number per spike in transgenic wheat by activating the starch and sucrose biosynthesis, flavonoid biosynthesis, lignin biosynthesis, and phytohormone signaling pathways. This work and its finding will deepen the understanding of wheat GNS development mechanisms and have high potential application value for wheat improvement.

## Data Availability

All data generated or analyzed during this study are included in this published article and its **Supplementary Information Files**. The raw RNA-Seq data in this study were submitted to the NCBI Sequence ReadArchive (accession numbers: PRJNA1206736).

## Supplementary material

Supplementary Figure 1 The expression levels of *TaPRX-2A*, *TaPRX-2B*, *TaPRX-2C* based on transcripts per kilobase million (TPM) values collected from the WheatOmics site.

Supplementary Figure 2 The count of all detected DEGs in *TaPRX-2A* overexpression lines, compared with WT plants.

Supplementary Figure 3 The relative expression of these genes in flavonoid biosynthesis, lignin biosynthesis, phytohormone, sucrose and starch biosynthesis pathways and TFs. The wheat gene *18SrRNA* was as an endogenous control. The relative expression of these genes was calculated by the cycle threshold (Ct) values using formula 2^–ΔΔCT^. Each experiment was conducted in three replicates and values are means ± SD.

Supplementary Figure 4 The interaction networks of differentially expressed genes in flavonoid biosynthesis, lignin biosynthesis, phytohormone, sucrose and starch biosynthesis pathways.

Supplementary Table 3 DEGs involved in flavonoid biosynthesis, lignin biosynthesis, phytohormone, sucrose and starch biosynthesis pathways.
