# Supplementary figures and images for: The Class III Peroxidase gene TaPRX-2A controls grain number per spike in common wheat (Triticum aestivum L.)
Source: Front Plant Sci. 2025 Feb 6;15:1501029. doi: 10.3389/fpls.2024.1501029 (PMC11839669; doi:10.3389/fpls.2024.1501029)

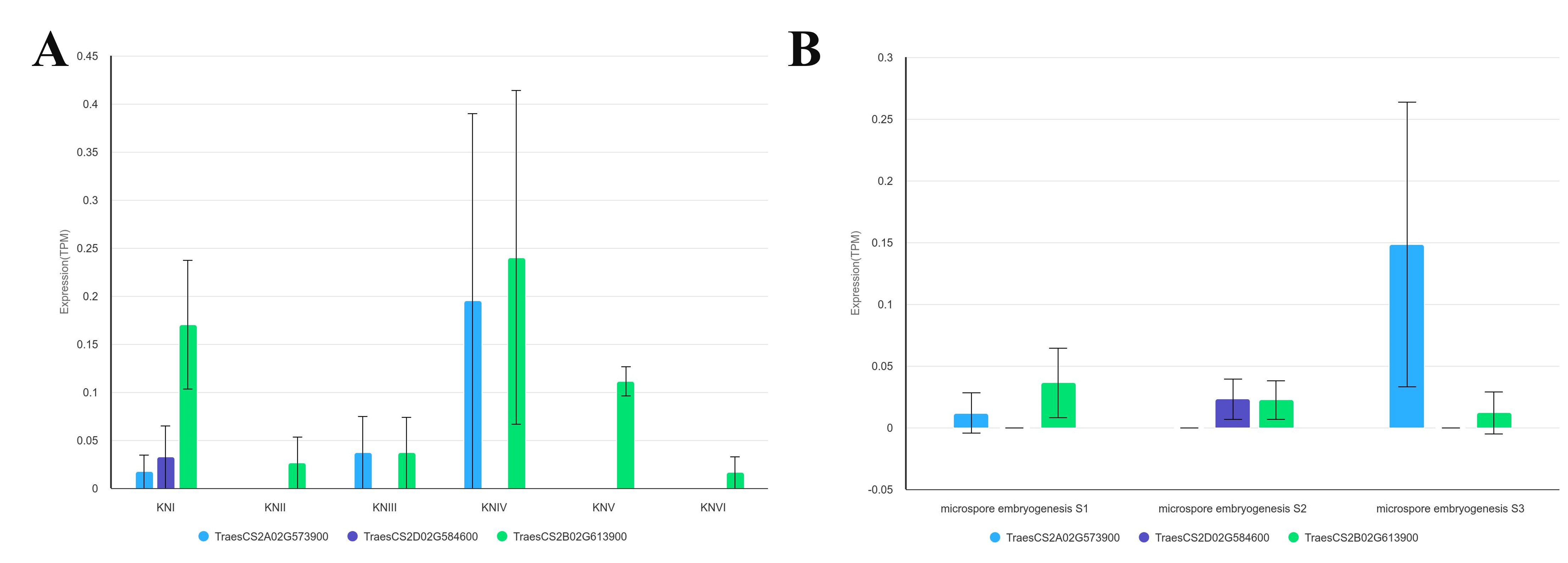

Supplement: Supplementary Figure 1 — The expression levels of TaPRX-2A, TaPRX-2B, TaPRX-2C based on transcripts per kilobase million (TPM) values collected from the WheatOmics site. [file Image1.jpeg]

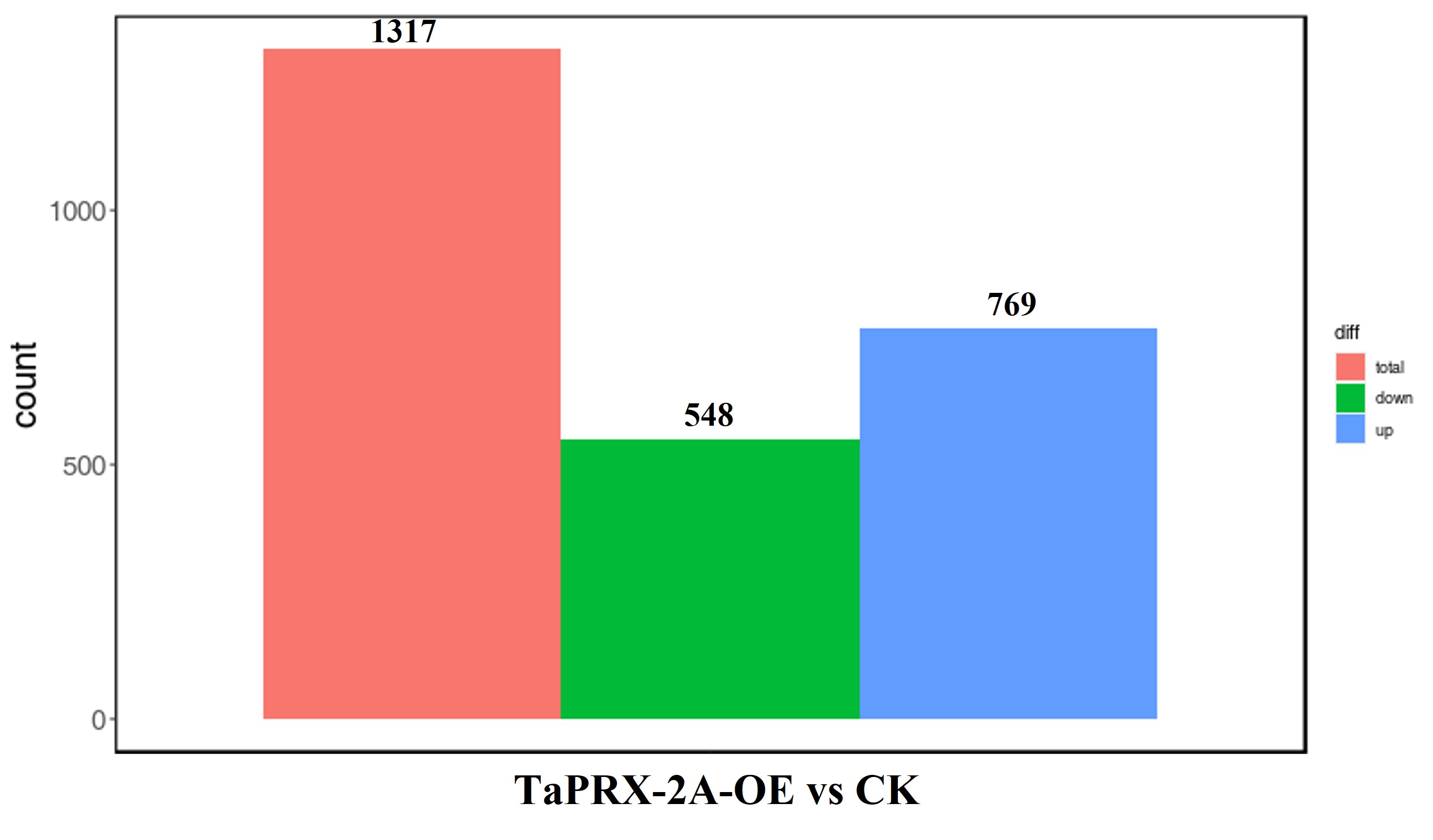

Supplement: Supplementary Figure 2 — The count of all detected DEGs in TaPRX-2A overexpression lines, compared with WT plants. [file Image2.jpeg]

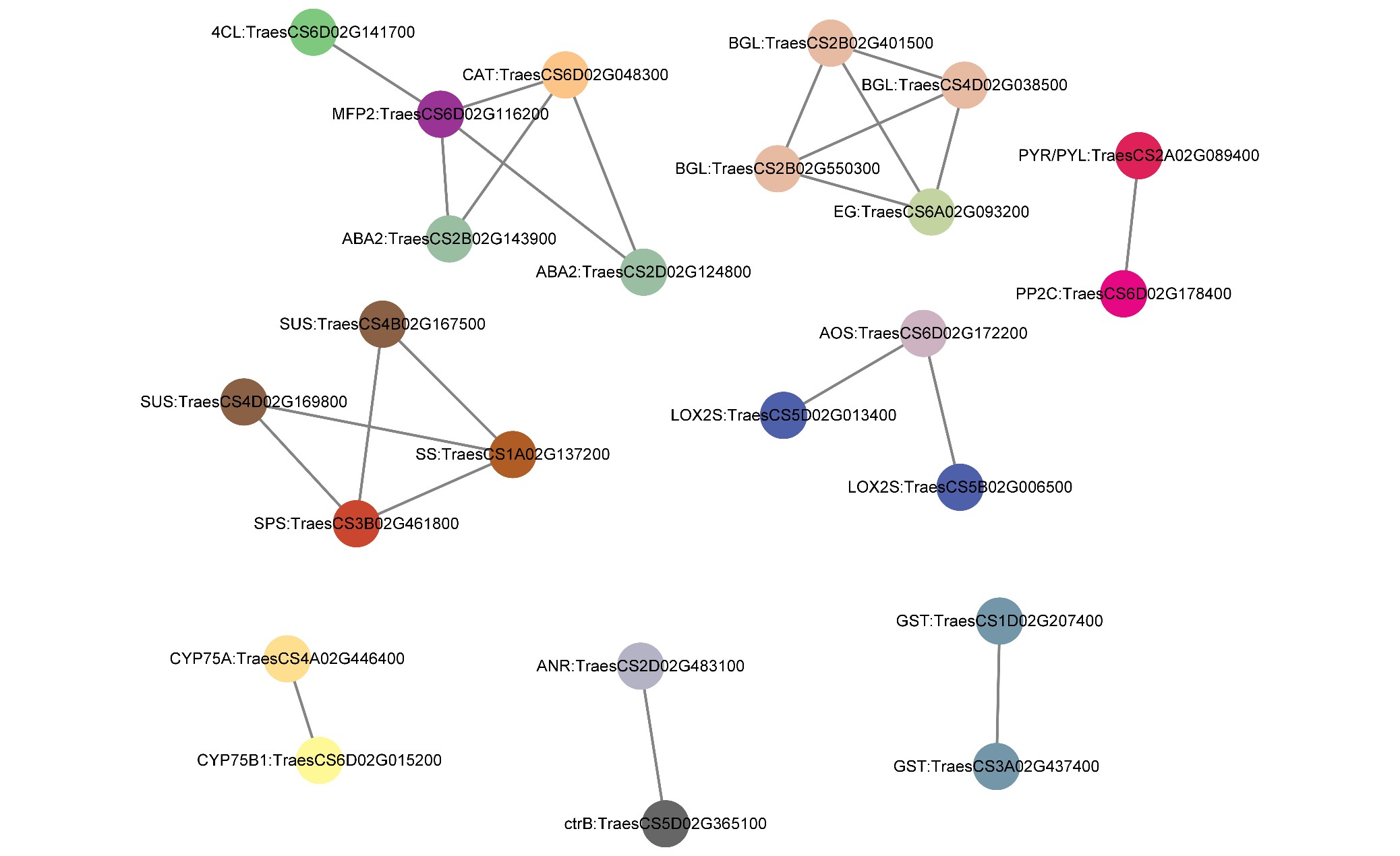

Supplement: Supplementary Figure 4 — The interaction networks of differentially expressed genes in flavonoid biosynthesis, lignin biosynthesis, phytohormone, sucrose and starch biosynthesis pathways. [file Image4.jpeg]
